# Numerical Analysis of the Process of Drawing Thin-Walled CuSn8 Alloy Tubes on a Cylindrical Plug

**DOI:** 10.3390/ma18122754

**Published:** 2025-06-12

**Authors:** Maciej Suliga, Sebastian Mróz, Piotr Szota, Mateusz Wasilewski, Konrad Jaroszewski

**Affiliations:** 1 Faculty of Production Engineering and Materials Technology, Czestochowa University of Technology, Armii Krajowej 19, 42-201 Czestochowa, Poland; sebastian.mroz@pcz.pl (S.M.); piotr.szota@pcz.pl (P.S.); 2 WIKA Polska SGF, Kawka 6, 87-800 Włocławek, Poland; mateusz.wasilewski@wika.com (M.W.); konrad.jaroszewski@wika.com (K.J.)

**Keywords:** tube drawing process, bronze, FEM, die geometry, temperature, stress and strain state, wear

## Abstract

The paper presents the results of FEM computer simulations of the drawing process on a cylindrical journal of thin-walled CuSn8 alloy tubes. This study demonstrates through FEM simulations that the drawing angle significantly affects the state of stress, strain and tool wear. Regardless of the geometry of the drawing die, greater wear was noted for the cylindrical plug. Increasing the angle of drawing die 2α from 6° to 38° contributed to a slight 5% increase in wear of the drawing dies and more than 80% increase in plug wear. Accelerated tool wear at high angles is to be associated with higher pipe pressures on the drawing die and plug. Inadequate selection of drawing geometry can cause additional material deformation effort and material fracture in the industrial drawing process of thin-walled tubes. After the drawing process, these tubes may also show non-uniform wall thickness. The optimum drawing angle for thin-walled tubes is 2α = 22°, for which about a 10% decrease in the drawing force was recorded.

## 1. Introduction

Precision thin-walled tubes are used in a wide variety of industrial sectors, including aerospace, defense and transportation, as well as in medical devices, measurement technology, the nuclear industry and many others [1,2,3,4]. In particular, applications for thin-walled tubing include fluid handling systems, metering equipment components and high-pressure installations, where extremely precise dimensional tolerances, high surface quality, high mechanical properties, homogeneity of material structure and resistance to corrosion and extreme environmental conditions are crucial, making them indispensable in the most demanding technical applications [5,6,7].

Tube processing can be carried out by various metal forming processes, among which the process of drawing deserves special attention. The characteristics of tube products vary depending on the forming process used and the drawing parameters, such as drawing angle, length of the calibration part, crush and friction coefficient [8,9,10,11,12]. These parameters not only affect the process of formation but also have a direct impact on the quality of the resulting tubes, including their geometry, mechanical properties and surface finish.

Drawing is one of the technologically significant processes of metalworking, involving drawing a tube through a conical, tapered die, called a drawing die, often while using a mandrel located inside the drawn tube [13,14,15,16]. The cold drawing process is widely used in the tube manufacturing industry because, in addition to the advantage of tight dimensional tolerances, it also offers a very high degree of flexibility in obtaining different tube sizes and the ability to modify their mechanical properties.

Basically, there are four methods of drawing tubes to reduce the external and internal diameters, namely free drawing (no mandrel involved), drawing on a cylindrical mandrel (mandrel in a fixed position), drawing with a long free mandrel and drawing with a floating mandrel. In all of the above-mentioned tube-drawing methods, the outer diameter of the tube is defined by the inner hole of the drawing die. The inner diameter (and wall thickness) of the tube is calibrated using one of three methods, with the exception of free drawing, for which the wall thickness is approximately constant and less controlled [17]. The process of drawing tubes using a fixed cylindrical mandrel is one of the methods most widely used in industrial production, as it allows to obtain tubes with high surface quality and precise dimensions, which is crucial for further use in the most demanding industrial and technical applications [1,17]. During plastic strain in the process of drawing the material, significant changes occur in its structure and mechanical properties, leading to its strengthening [18,19]. When a significant reduction in cross-section is required, the drawing process is carried out in stages, with recrystallization annealing between the respective stages, so that the material’s ductility is restored. With successive drawing, the strain resistance increases, leading to an increase in material effort and tube breakage during the drawing process. Hence, it is necessary to optimize the drawing process, and the minimum drawing force can be the optimization criterion. The frequency of intermediate heat treatments depends on the rate of material strengthening. For example, under industrial conditions, the total squish, when drawing on a plug of cylindrical copper alloy tubes, usually does not exceed 50%.

Drawing as a plastic processing method is widely used in industry. This method is used in the drawing processes of carbon steel, stainless steel, metals and non-ferrous metal alloys, including copper, aluminum, magnesium and magnesium alloys [20,21,22,23,24]. While the literature quite widely describes the drawing processes of medium and thick-walled steel and copper pipes, there are few works describing the processes of manufacturing thin-walled pipes from non-ferrous metal alloys with a wall thickness below 2 mm. For example, in [25], the drawing process of copper pipes with an external diameter of 65 and a wall thickness of 5.5 mm is described. Increasing requirements for the quality of pipes and pipe products, especially from non-ferrous metal alloys, force manufacturers to use increasingly new methods of designing pipe drawing technologies. Data presented in the literature [26] confirm that the use of numerical methods in the design of pipes significantly shortens the design time of new products, and the obtained results are a valuable supplement to experimental research.

In addition to carbon steels, stainless steels or aluminum alloys used in the manufacture of thin-walled tubes, copper alloys are an important class of materials, distinguished by favorable combination of mechanical, physical and chemical properties with respect to raw material costs as well as manufacturing process costs. The most common copper alloys are primarily bronzes and brasses [27]. Tin bronzes (CuSn) such as CuSn6 and CuSn8 are characterized by excellent abrasion resistance and corrosion resistance in aquatic and marine environments. CuSn12 alloy, with a higher tin content, demonstrates increased resistance to acid corrosion, hence its frequent use in the chemical industry. In turn, the CuSn8P alloy is characterized by increased fatigue resistance and better tribological properties. Brasses (CuZn), including plain brass CuZn37 and lead brasses CuZn40Pb2 and CuZn39Pb3, are particularly attractive due to their high ductility, ease of machining and corrosion resistance. The listed properties, as well as low cost, make them widely used not only in the production of tubes, including heating tubes but also in the production of fittings, valves and machine parts. Special brasses, such as CuZn15Al and CuZn20Sn, are characterized by increased abrasion and corrosion resistance.

The available technical literature provides the basis for designing tubular products with standard dimensions and enables an understanding of the basic mechanisms inherent in the drawing process [27,28]. Nevertheless, it seems that the number of experimental research studies, as well as studies combining experimental and analytical or numerical approaches, is still insufficient, especially in the context of precision thin-walled tubes meeting the highest requirements. In most cases, due to the high cost of tools, optimization of the tube drawing process is based on empirical formulas, which give an overall view of the drawing process. A much better solution is to combine experimental studies with computer simulations. Numerical modeling enables detailed analysis of material flow distribution, condition of stresses and strains, as well as wear of tools in the process of drawing precision tubes.

The study analyzed drawing on a cylindrical mandrel of CuSn8 bronze tubes for a variable geometry of the drawing die with fixed dimensions of the cylindrical mandrel. Experimental studies determined the drawing force, while computer simulations made it possible to analyze stress, strain and temperature distributions, as well as tool wear.

## 2. Materials and Methods

### 2.1. Computer Simulation

As part of the theoretical research conducted, computer simulations were performed to determine the process of drawing tubes on a cylindrical plug made of CuSn8 bronze. The use of numerical modeling made it possible to determine the effect of drawing die geometry on drawing parameters, i.e., temperature, strain, stress, drawing force, tool wear. The analysis of these parameters allowed the selection of shape parameters of the tools that guarantee obtaining tubes with the assumed characteristics while having a long tool life.

Computer simulations of the tube drawing process were carried out using an elastoplastic model of material strain, in which the mechanical condition of the strained material is described using the Norton–Hoff law [29,30]:

(1) $$S_{ij}=2K(T,\dot{\bar{\varepsilon }},\bar{\varepsilon })(\sqrt{3}\dot{\bar{\varepsilon }}{)}^{m-1}{\dot{\varepsilon }}_{ij}$$

where S_ij_—stress tensor deviator, $\dot{\bar{\varepsilon }}$—strain rate intensity, ${\dot{\varepsilon }}_{ij}$—strain rate tensor, $\bar{\varepsilon }$—strain intensity, T—temperature, K—consistency depending on plasticizing stress σ_p_, m—coefficient characterizing metal strain (0 < m < 1).

Friction model:

Frictional conditions at the metal–tool interface are described using the Coulomb friction model and Treska friction model. The differential equation describing temperature changes for transient heat flow is used for determining the temperature change.

(2) $$\tau_{j}=\mu \cdot \sigma_{n}\;\mathrm{dla}\;\mu \cdot \sigma_{n}<\frac{\sigma_{0}}{\sqrt{3}},$$

(3) $$\tau_{j}=m\frac{\sigma_{0}}{\sqrt{3}}\;\;\mathrm{dla}\;\mu \cdot \sigma_{n}>m\frac{\sigma_{0}}{\sqrt{3}},$$

where *τ_j_*—vector of unit friction forces, *σ*_0_—base stress, *σ_n_*—normal stress, *μ*—friction coefficient, *m*—friction factor.

Thermal model:

To determine the temperature field, a differential equation describing the temperature changes during unsteady heat flow is used. This is a quasi-harmonic equation of the form:

(4) $$\frac{\partial }{\partial x}\left(k_{x}\frac{\partial T_{s}}{\partial x}\right)+\frac{\partial }{\partial y}\left(k_{y}\frac{\partial T_{s}}{\partial y}\right)+\frac{\partial }{\partial z}\left(k_{z}\frac{\partial T_{s}}{\partial z}\right)+\left(Q-c_{p}\rho \frac{\partial T_{s}}{\partial t}\right)=0$$

In this equation, kx, ky and kz are functions of the distribution of anisotropic thermal conductivity coefficients in the x, y and z directions. Ts is a function that describes the temperature in the considered zone, Q is a function of the distribution of the rate of deformation heat generation, cp is a function of the distribution of the specific heat of the metal and ρ is a function of its density distribution.

The combined boundary conditions of the second and third kind were adopted as boundary conditions, which can be written in the form:

(5) $$k_{x}\frac{\partial T_{s}}{\partial x}l_{x}+k_{y}\frac{\partial T_{s}}{\partial y}l_{y}+k_{z}\frac{\partial T_{s}}{\partial z}l_{z}+q+\alpha_{k}T_{s}=0$$

In this equation, lx, ly and lz are the directional cosines of the normal to the surface of the strip, q is the heat flow rate on the surface of the cooled zone and αk represents convective losses. Equation (5) and boundary condition 0 uniquely define the heat exchange during the modeling of the rolling process. The tools in the drawing process were assumed to be rigid.

Tool wear occurs during metal forming. The most common type of wear in these processes is abrasive wear [31]. The Archard model is often used for describing abrasive wear [32]. The model assumes that under abrasive wear conditions, the volume of material V_z_ separated from a unit area of the tool is directly proportional to the normal stress σ_n_ acting on the tool surface and the friction path L_t_ and inversely proportional to the hardness of the material H being worn (in this case, the hardness of the tool). The model can be written in the following form:

(6) $$V_{Z}=k_{wear}\frac{\sigma_{n}L_{t}}{H}$$

where *k_wear_*—wear factor, *σ_n_*—normal stress, *H*—hardness, *L_t_*—length of contact with die.

Equation (6) can be presented in integral form for solution using an FEM-based algorithm:

(7) $$V_{Z}=k_{wear}\int_{0}^{t}\frac{\sigma_{n}\cdot v_{s}}{H\left(T\right)}dt$$

where v_s_—the tangential sliding speed of the metal on the tool surface, t—time, H(T)—hardness of the tool at the specified temperature.

In the model used in computer program, Equation (6) was simplified to the following form:

(8) $$W=\int_{0}^{t}\sigma_{n}\cdot v_{s}dt$$

where W—unit work of friction force.

The computer program can also be used for calculating the force parameters of the mandrel tube drawing process. In this paper, the value of the total pressure force (drawing force) was determined based on the results of computer simulations. The total force of the metal on the drawing die, and mandrel was determined by numerical integration of the stress normal to the tool surface.

To the model described by Equation (1), a model of the properties of CuSn8 bronze was introduced in the form of a function describing the stress from strain and in the form of a table. The models introduced took into account Young’s modulus and the physical properties of bronze.

**Figure 1:**
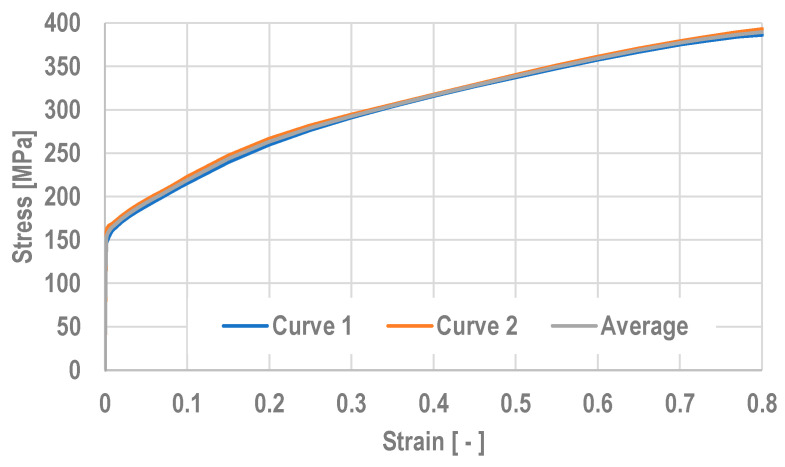
Curves obtained in static tensile testing of CuSn8 bronze alloy specimens.

Determination of the value of the flow stress σ_f_ of the tested bronze is very significant when designing the plastic forming processes. The correct determination of bronze properties in the form of stress–strain diagrams provides increased accuracy of calculations when using empirical formulas, as well as for numerical calculations that use the finite element method. The plastometric tests were carried out using a ZWICK Z/100 testing machine (ZwickRoell, Ulm, Germany). During the tensile testing of the specimens, the testXpert program was used to control the ZWICK Z/100 testing machine, which enabled to determine the characteristic strength properties of the tested steel: yield strength—YS, tensile strength—UTS and total elongation TEL%. Static tensile tests were conducted for flat specimens 3 mm wide and 1 mm thick. Samples were taken from a supplied pipe with a diameter of 18.5 mm and a wall thickness of 1 mm. The length of the test specimens was 200 mm. Figure 1 and Table 1 show the results of the tensile tests. The tests were conducted for 2 samples, after which the results obtained were averaged.

It can be seen from Table 1 that the values declared by the manufacturer are higher than the values obtained during tensile tests. At the same time, it was noted that the elongation significantly exceeds the minimum values declared by the manufacturer, which is favorable due to the plastic forming process during cold drawing of tubes.

Based on the results of the approximation of the tensile curves, the coefficients of the stress function (9) were selected and used when conducting computer simulations for the tensile process.

(9) $$\sigma_{f}=K\cdot \varepsilon^{m_{1}}\cdot \exp^{\left(\varepsilon \cdot m_{2}\right)}\;$$

where σ_f_—flow stress, ε—true strain, K, m_1_ ÷ m_2_—function coefficients.

After approximating the results of the tensile tests, the coefficients for Equation (9) were determined. The values of these coefficients are shown in Table 2. Young’s modulus was calculated using the tangent method. The value of Young’s modulus for the bronze alloy tested was 131,829 MPa.

The data describing the flow curves of the tested bronze alloy was entered into the computer program used in this study in the form of Table 3, in which, as in Equation (9), the values of the flow stress depend on the value of the actual strain.

The properties of the tested alloy were entered into the computer simulation in the form of a table; the graphical presentation is shown in Figure 1.

The application of the computer program using the thermo-mechanical models contained therein requires defining the boundary conditions that determine the correctness of numerical calculations. Therefore, the properties of the tested bronze alloy, the friction conditions and the kinetic and thermal parameters describing the drawing process have a significant impact on the calculation results.

The boundary conditions include the properties of the tools and the properties of the material being strained: flow stress, density, specific heat, temperature, heat capacity, heat transfer coefficients, friction, etc.

For the theoretical analysis of the process of drawing tubes on the plug, the following input data was taken: tool temperature—20 °C; ambient temperature—20 °C; tube temperature—20 °C, friction coefficient—0.05; friction factor—0.1; coefficient of heat transfer between material and tool—α_narz_ = 20,000 [W/(K·m^2^)]; coefficient of heat transfer between material and air—α_pow_ = 100 [W/( K·m^2^)], drawing rate—0.7 m/s.

In order to carry out numerical calculations, it is necessary to prepare models of the tools and the batch tube. These models were designed using CAD software Solidworks 2025 Dassault System. In the study, the models were surface objects whose surfaces were then transformed into a finite element mesh (mesh generation process). The computer program uses models made from a finite element mesh, the base element of which is a triangle. A surface 3D mesh was used for tools (drawing die, mandrel, handle), while a spatial mesh based on the surface mesh was generated for drawn items (tube). The finished spatial mesh in the strained object was constructed of tetrahedral elements, the size of which was determined by the average edge length of the element. An important part of preparing the computer simulation was development of an appropriately sized mesh. The size of the mesh significantly determines the accuracy of the calculations, as well as the rate of the calculations carried out. It becomes necessary to determine the optimal size of mesh elements by choosing a balance between calculation accuracy and calculation time. The computer program allows generation of mesh, for example, on corners, on curves, in areas with complex shapes and small dimensions, by defining mesh compaction zones called mesh boxes. The zone-based mesh compaction tool was useful for numerical modeling, but it did not completely eliminate the problem of sizing the finite element mesh. The number of elements in the strained object also depends on the length of the tube during the simulation of drawing. The optimal mesh size and tube length were determined based on the authors’ experience gained from using the computer program to simulate plastic processing. According to the authors, the size of the mesh should be between 0.05 and 0.07 of the thickness of the drawn tube, and the length of the tube adopted in the simulation should be between 2 and 3 of the length of the strain basin.

**Figure 2:**
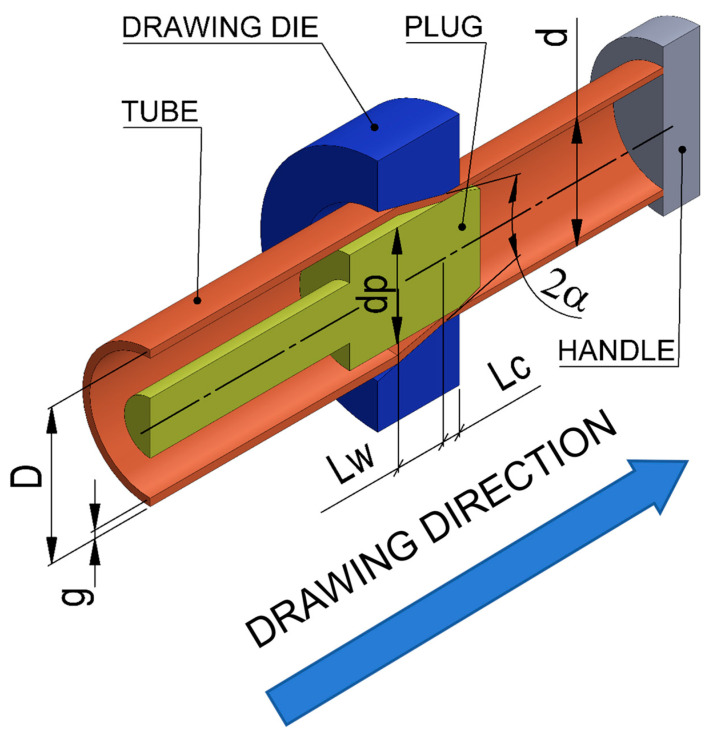
Surface model of tools and tube.

Due to the fact that the tube drawing process is axisymmetric during the simulation of the drawing process, only 1/60th of the cross-section of the tools and tube was used to increase the rate and accuracy of the calculations. Figure 2 presents example models of tools and tubes.

Computer simulations were performed for tube drawing processes on a cylindrical plug differing in the drawing angle and in the length of the calibration part (Table 3).

### 2.2. Experimental Studies

**Figure 3:**
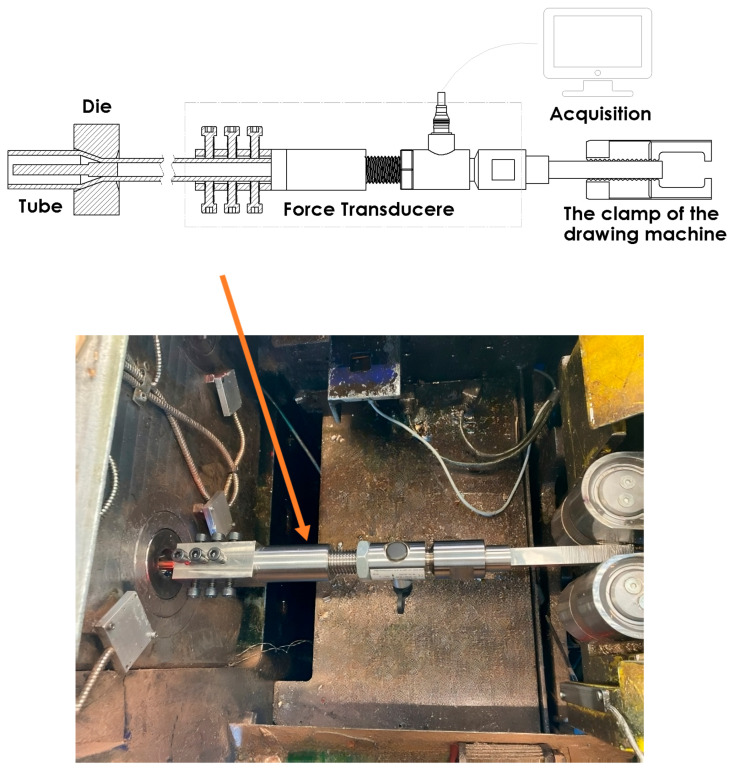
Drawing force measurement system.

In order to verify the results of the computer simulations in the paper, an experimental measurement of the drawing force of pipes on a cylindrical plug was carried out. The force was measured for a single drawing, for drawing dies of different geometries according to Table 3. The initial dimensions of the tube were 18 × 1 mm (diameter × wall thickness), while after drawing, they were 15.200 × 0.775 mm. The drawing force was measured under industrial conditions on a bench-drawing machine under dynamic conditions using a Tecsis F2304 force transducer (WIKA Instruments Ltd., Edmonton, AB, Canada). Mineral oil was used as a lubricant for the drawing process. Figure 3 shows the measurement system. The bench configuration ensured that the impact of mechanical and electrical interference was minimized, and the signal was digitally filtered to eliminate noise. Three drawing force measurements were carried out for each drawing variant.

## 3. Results

**Figure 4:**
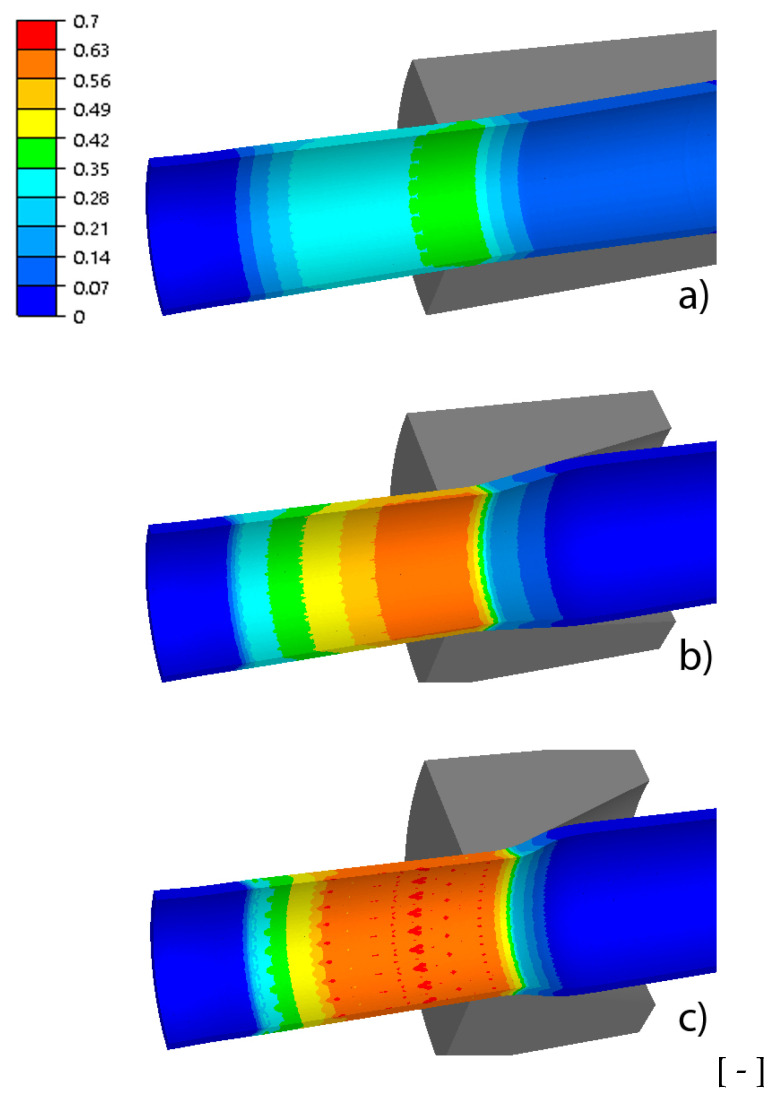
Comparison of the distribution of the plastic equivalent strain of the tube for different drawing angles, where (**a**) variant I—2α = 6°, (**b**) variant III—2α = 22°, (**c**) variant V—2α = 38°.

**Figure 5:**
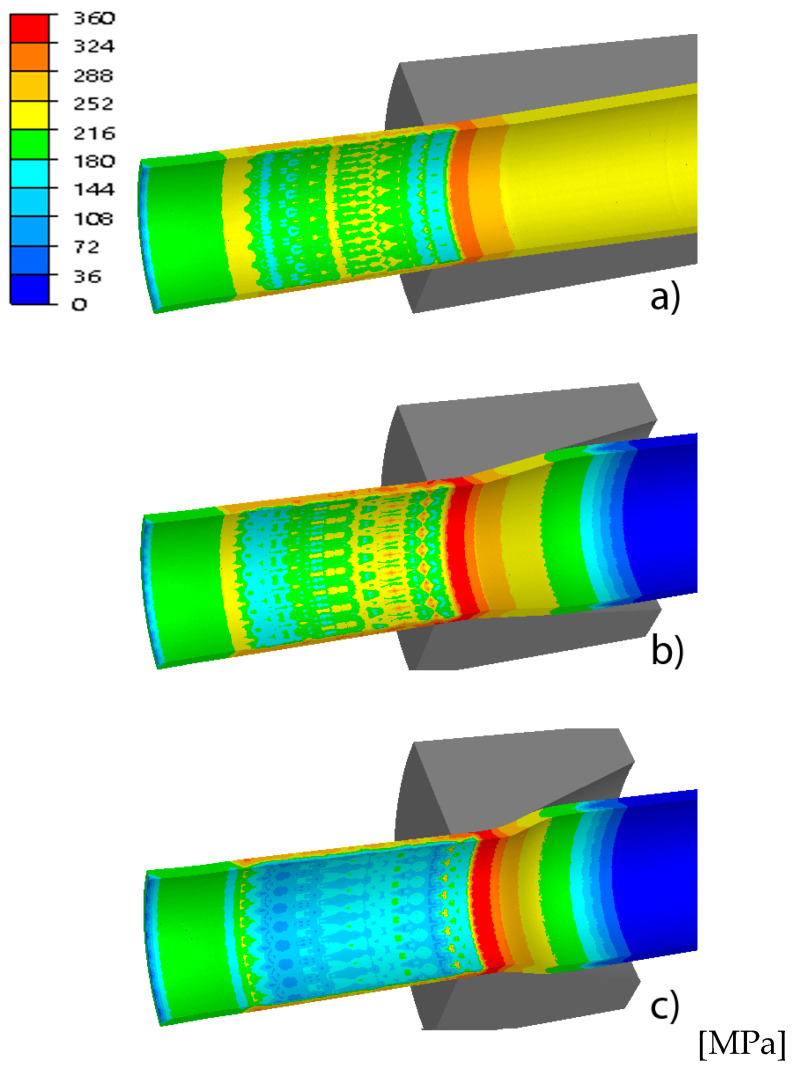
Comparison of the distribution of equivalent stress of the tube for different drawing angles, where (**a**) variant I—2α = 6°, (**b**) variant III—2α = 22°, (**c**) variant V—2α = 38°.

**Figure 6:**
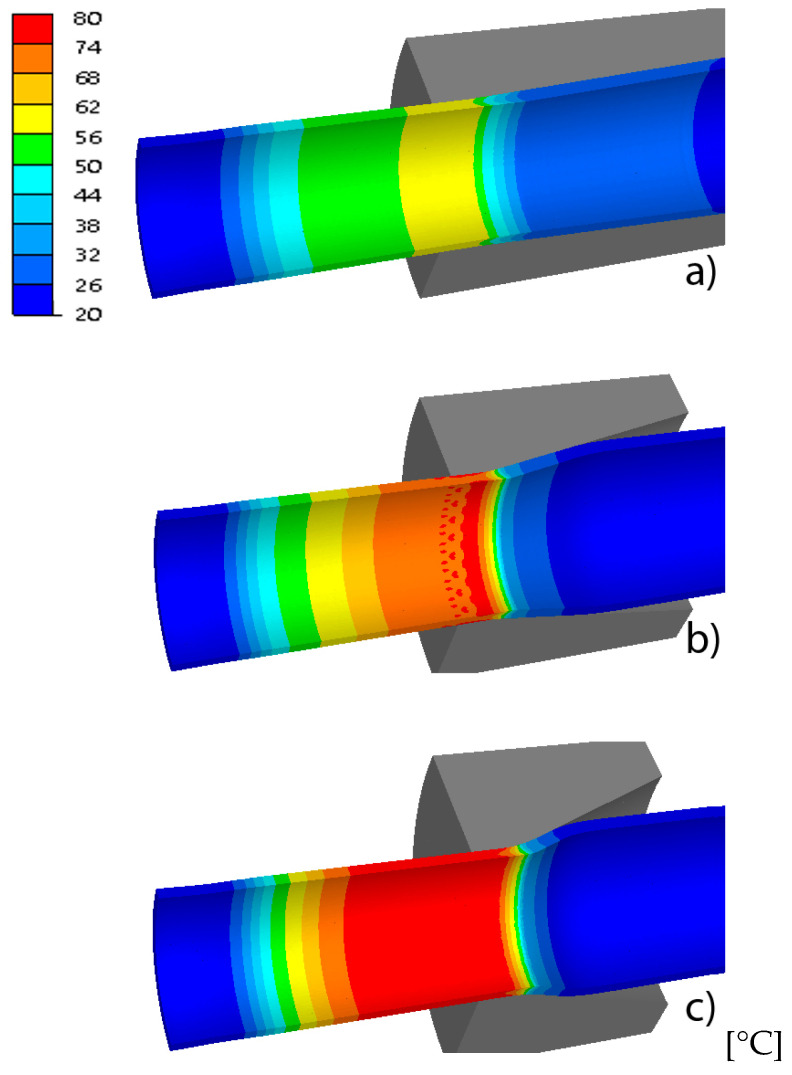
Comparison of the distribution of temperature of the tube for different drawing angles, where (**a**) variant I—2α = 6°, (**b**) variant III—2α = 22°, (**c**) variant V—2α = 38°.

**Figure 7:**
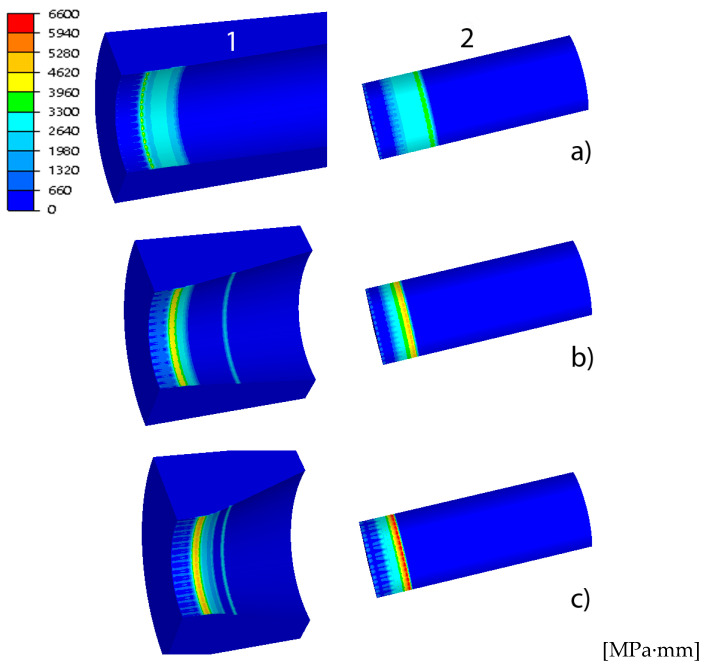
Comparison of the distribution of tool wear for different drawing angles, where (**a**) variant I—2α = 6°, (**b**) variant III—2α = 22°, (**c**) variant V—2α = 38° (1-drawing die, 2-cylindrical plug).

The basic parameters of the process of drawing tubes on a cylindrical plug include drawing force, strain and stress intensity, temperature and tool wear. These parameters determine the tube’s tensile strength, surface quality, geometric dimensions, mechanical properties and depend on the geometry of the drawing dies, among others. Figure 4, Figure 5, Figure 6 and Figure 7 show examples of the distributions of strain intensity, stress intensity, temperature, unit work of frictional forces on the drawing die, and the unit work of frictional forces on the cylindrical plug.

The distributions of equivalent stress and strain, temperature and tool wear, shown in Figure 4, Figure 5, Figure 6 and Figure 7, confirmed the influence of drawing die geometry on the tube drawing process. Figure 4, Figure 5, Figure 6 and Figure 7 show that the tubes drawn in drawing dies with an angle of 2α = 38° compared to tubes drawn with an angle of 2α = 6° are characterized by greater reinforcement caused by an increase in non-dilatational strains.

**Figure 8:**
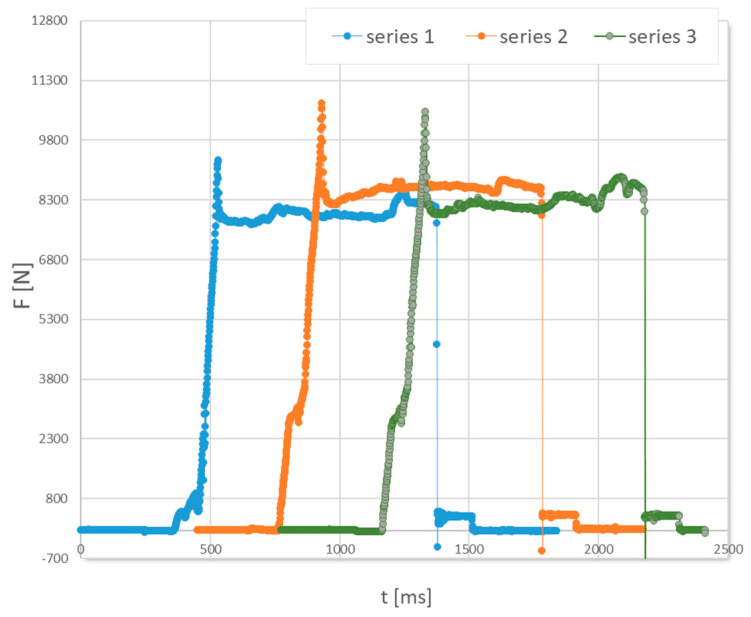
Change in the drawing force during passage of the tube through the drawing die (three measurements), 2α = 22°.

**Figure 9:**
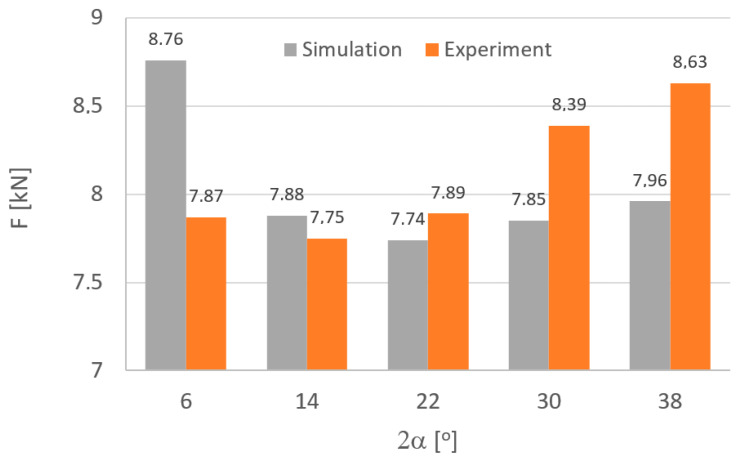
Comparison of the average values of drawing forces obtained in numerical modeling and experimental studies.

To verify the data obtained, the paper compares the values of forces measured during the drawing process under industrial conditions with those obtained from computer simulations. Figure 8 shows an example of the course of the drawing force when the tube is pulled through the drawing die (variant III). In the initial phase, there is a very rapid increase in force, which should be associated with the onset of strain of the tube. After drawing 20 mm of the material, the drawing force was noted to be stabilizing; for example, the average value of the drawing force in the stabilized process for variant IV was F = 8395 N. Then, for the other drawing variants, the force value for the stabilized drawing process was read and recorded. The average force values are shown in Figure 9.

The data in Figure 9 shows that the differences in the values of forces obtained from experiments and simulations range from 1 to 11%, depending on the variant. The resulting differences should be associated, among others, with dimensional deviations of the tools. Small differences, of the order of a few hundredths of a millimeter, were found between the nominal and measured dimensions. In numerical calculations, constant friction conditions are assumed, while during the actual process, the friction conditions may change, especially at different drawing angles. Hence, in the angle range of 12–22°, the differences between the measured values and those obtained from simulation are less than 2%. It is extremely difficult to determine lubrication efficiency, so in order to be able to evaluate the effect of drawing parameters, the same friction conditions were assumed.

**Figure 10:**
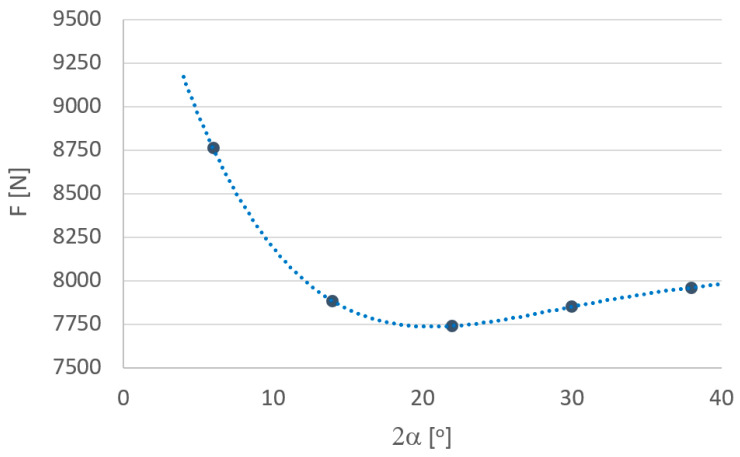
Effect of drawing angle 2α on the drawing force.

**Figure 11:**
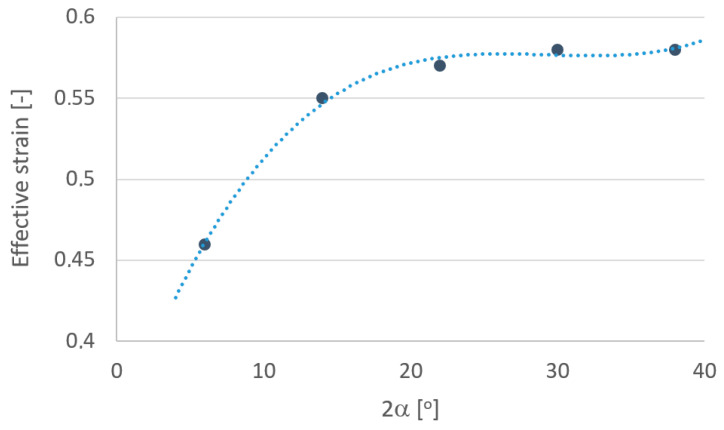
Effect of drawing angle 2α on the intensity of strain of the tube.

**Figure 12:**
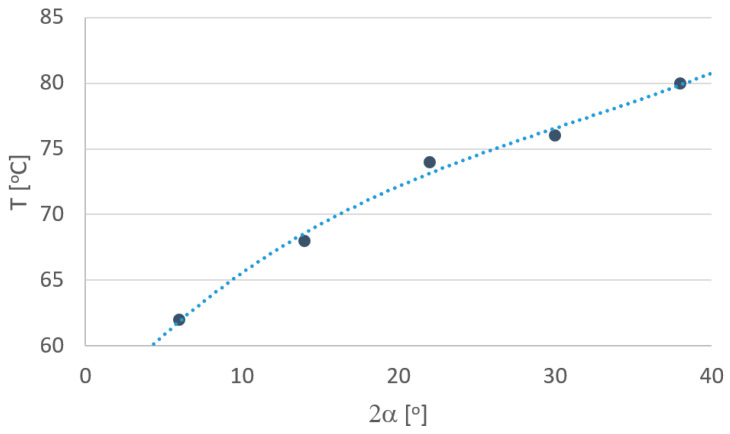
Effect of drawing angle 2α on the temperature of the tube.

**Figure 13:**
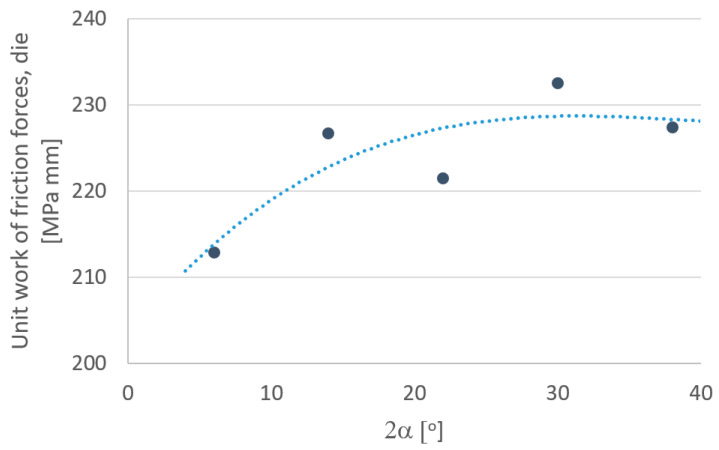
Effect of drawing angle 2α on the unit work of frictional forces—the drawing die.

**Figure 14:**
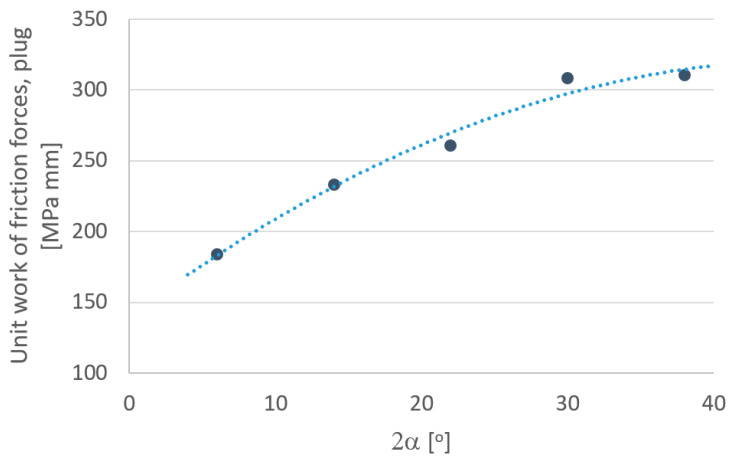
Effect of drawing angle 2α on the unit work of frictional forces—plug.

**Figure 15:**
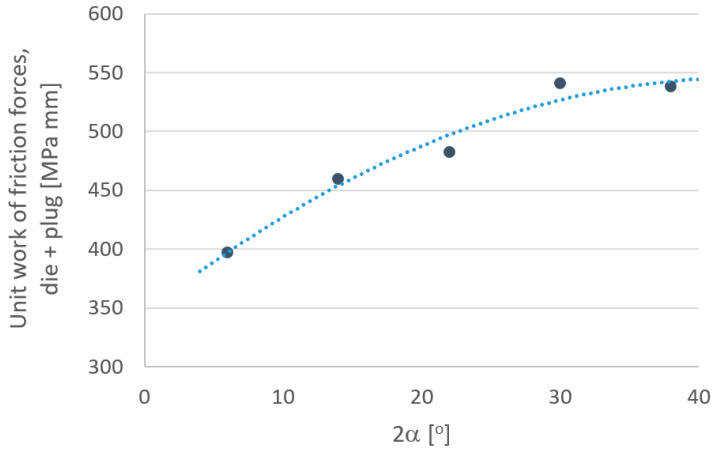
Effect of drawing angle 2α on the unit work of frictional forces—drawing die + plug.

In order to more fully analyze the research results, based on computer simulations, graphs were drawn defining the influence of individual parameters depending on the angle of the drawing die (Figure 10, Figure 11, Figure 12, Figure 13, Figure 14 and Figure 15).

Analysis of the test results shown in Figure 10 revealed that the drawing force as a function of the drawing angle is not a linear function. Unlike the process of drawing solid products (wires, rods), the process of drawing tubes involves a much more complex state of stresses and strains. In the process, there is a reduction in the diameter of the tube with a simultaneous reduction in its thickness. In the first stage, there is a hollow drawing zone. In this area, there is a reduction in the diameter of the tube with slight thickening, similar to free drawing. For the drawing conditions analyzed, it was an average of about 0.05 mm, with greater wall thickening noted for small drawing angles. This contributed to an increase in the total strain of the tube. In the second stage, there is a reduction in wall diameter and thickness. At this stage, the tube elongation coefficient resulting from a reduction in the wall varies, and its exact value depends on how the wall is thickened in stage I. In turn, in the last, third stage, there is a tube calibration zone. The drawing angle affects these zones, the resulting drawing force is the resultant of the forces in these three zones. In addition, an increase in the drawing angle causes, on the one hand, a decrease in the length of the tube/drawing die interface at which frictional forces act, and on the other, it contributes to an increase in the pressure of the tube on the drawing die. Changing the angle of drawing can cause either an increase or a decrease in drawing force. For the analyzed tube drawing process, the minimum values were recorded for the drawing angles of ca. 2α = 24°, Figure 10. Therefore, according to the authors, the optimal drawing angle for CuSn8 alloy precision tubes is 2α = 24°.

The data shown in Figure 11 shows that the largest increase in strain intensity occurs in the range of angles of 2α = 6–14°. During the drawing process, a phenomenon takes place of braking of the surface layers of the tube as a result of friction on the tube/drawing die surface. As a result, the grains are not only elongated in line with the direction of strain but also twist by a certain angle, contributing to an increase in total strain. In the literature, this additional strain is called a non-dilatational (redundant) strain. The increase in the angle of drawing caused an increase in the non-dilatational strain and, consequently, an increase in the intensity of the strain and a decrease in the ductility of the tubes. Tubes with reduced ductility tend to break during further plastic operations, such as bending, spring forming, etc.

Another parameter that characterizes the drawing process is the temperature of the tube. In the drawing process, there is a gradual heating of the material along the length of the tube/drawing die interface. The factor that affects the amount of heat generated in the drawing process is the drawing angle. The temperature values shown in Figure 12 refer to the temperature of the tube when leaving the drawing die. Simulation results showed that in the range of angles of 2α = 6–38°, increasing the drawing angle resulted in an increase in the temperature of the tube. The maximum differences in temperature values among the analyzed variants were ca. 20%. The increase in the temperature of drawn tubes with large drawing angle values should be associated, among others, with higher drawing die and plug pressure on the tube

An analysis of tool wear based on the unit work of frictional forces of the drawing die and tube showed that the angle of the drawing significantly affects the degree of wear of the drawing die and plug. Increasing the drawing angle in the analyzed range resulted in an increase of up to 5% in drawing die wear and a more than 80% increase in cylinder plug wear. Such a significant increase in the wear of the cork when drawing in drawing dies with a large drawing angle should be associated with an increase, for this variant, of drawing with the unit pressure of the inner part of the tube on the surface of the cork. By averaging the results obtained for the drawing dies and plugs, it can be assumed that increasing the angle of the drawing increases the wear of the drawing die and plug. The highest total tool wear (drawing die + plug) was recorded for the angles of 2α = 30–38°. Thus, optimization of the tube drawing process in terms of tool wear is a complex issue and results from many factors, such as tube geometry, work of friction and plastic strain, temperature and pressure.

## 4. Conclusions

The conducted research confirmed that optimization of the process of drawing precision thin-walled tubes should use the criterion of the lowest drawing force. The optimal drawing angle for CuSn8 alloy precision tubes is 2α = 22°. According to the authors, the indicated optimal drawing angle can be generalized to the drawing process of pipes with similar geometry and chemical composition.

Analysis of the state of strain has shown that increasing the angle of the drag leads to an increase in the non-dilatational strain and, consequently, to an increase in the intensity and inhomogeneity of the strain on the cross-section of the tube. A material with such characteristics has a reduced capacity for further plastic strain during bending and coiling operations, for example, into a spring (precision industry—measuring equipment). Thus, a second, additional optimization criterion for the tube drawing process can be the criterion of minimum redundant strain.

In addition to the criterion of minimum drawing force and redundant strain, the third optimization criterion for the process of drawing precision tubes is the criterion of minimum tool wear. Increasing the drawing angle in the range of 6–38° resulted in a 5% increase in drawing die wear and a more than 80% increase in cylinder plug wear. Such a significant increase in the wear of the cork when drawing in drawing dies with a large drawing angle should be associated with an increase, for this variant, of drawing with the unit pressure of the inner part of the tube on the surface of the cork. The observed increase in tool wear at higher drawing angles is attributed to elevated contact pressures between the tube and the tool surfaces. Such a significant acceleration of plug wear can contribute to the deterioration of the tube surface—the possibility for cracks and seizures to appear, as well as to an increase in its dimensional deviations.

## Figures and Tables

**Table 1:** Averaged results of mechanical properties for the tested bronze alloy CuSn8.

Material	YS/ MPa	UTS/ MPa	TEL/ %
Tensile test	155	387	80

**Table 2:** Values of K, m_1_ and m_3_ parameters used for determining *σ_f_* of the tested CuSn8 bronze alloy.

Material	*K*	*m_1_*	*m_2_*
CuSn8	280	0.1	0.46

**Table 3:** Designation of drawing variants.

Variant	Drawing Angle 2α [^o^]	Length of the Calibration Part Lc [mm]	Length of the Calibration Part Lc [%]	Drawing Die Diameter D [mm]	Cylindrical Plug Diameter dp [mm]
I	6	4	26	15.20	13.65
II	14	4	26	15.20	13.65
III	22	4	26	15.20	13.65
IV	30	4	26	15.20	13.65
V	38	4	26	15.20	13.65

## Data Availability

The original contributions presented in this study are included in the article. Further inquiries can be directed to the corresponding author.
